# Respiratory Illness and Diarrheal Disease Surveillance in U.S. Military Personnel Deployed to Southeast Asia for Military Exercises from 2023–2025

**DOI:** 10.3390/tropicalmed10120353

**Published:** 2025-12-17

**Authors:** Sidhartha Chaudhury, Paphavee Lertsethtakarn, Piyawan Chinnawirotpisan, Nattaya Ruamsap, Worachet Kuntawunginn, Chadin Thongpiam, Kingkan Pidtana, Kittijarankon Phontham, Saowaluk Wongarunkochakorn, Montri Arsanok, Kamonporn Poramathikul, Parat Boonyarangka, Paksathorn Kietsiri, Wilawan Oransathit, Siriphan Gonwong, Patcharawalai Wassanarungroj, Panida Nobthai, Nuanpan Khemnu, Thipwipha Phonpakobsin, Wudtichai Manasatienkij, Joonlasak Khajohn, Chonthicha Klungthong, Nillawan Buathong, Sabaithip Sriwichai, Siriporn Sornsakrin, Umaporn Suksawad, Susie Leonardia-Santiago, Maria Theresa Valderama, John Mark Velasco, Paula Corazon Diones, Matthew Pascual, Chris Mahabir, Kathryn A. McGuckin, Daniel M. Boudreaux

**Affiliations:** Walter Reed Army Institute of Research—Armed Forces Research Institute of Medical Sciences, Bangkok 10400, Thailand

**Keywords:** respiratory illness, diarrheal disease, military, surveillance

## Abstract

The Indo-Pacific region hosts several annual military exercises that involve the deployment of thousands of U.S. and partner-nation military personnel. Respiratory and diarrheal diseases pose a significant health risk to exercise participants and represent a substantial portion of medical encounters and lost duty days. We conducted surveillance for respiratory and diarrheal illness at the Cobra Gold and Balikatan military exercises in Thailand and the Philippines from 2023–2025. Through coordination with health providers in the field, military personnel that reported acute symptoms were asked to provide a nasopharyngeal swab or stool sample. These samples were transported to a field lab and tested by PCR for common respiratory and diarrheal pathogens. Follow-up analyses included bacterial culture, antimicrobial susceptibility testing, and viral whole-genome sequencing. From 84 respiratory and 61 diarrheal samples analyzed, we found that respiratory illness was primarily attributed to rhinoviruses/enteroviruses (23%), common coronaviruses (21%), and SARS-CoV-2 (11%) while diarrheal disease was attributed to a high rate of diarrheagenic *E. coli* (73%) and norovirus (20%), followed by *Salmonella* spp. (18%) and *Campylobacter* spp. (13%). Our findings highlight the distinct etiologies of respiratory and diarrheal disease in military field settings and demonstrate the feasibility of conducting real-time infectious disease surveillance in operational environments.

## 1. Introduction

Infectious diseases have significantly impacted military operations throughout history resulting in disease non-battle injuries (DNBIs) that temporarily or permanently remove military personnel from the battlefield. Militaries operating in foreign countries under austere conditions are particularly susceptible to infectious diseases due to close-quarter living, suboptimal sanitation, and immunological naivety to endemic pathogens [1]. Examples of infectious diseases abound in military history including typhus and dysentery in 18^th^ and 19^th^ century wars throughout Europe and America [2,3], Dengue fever during the Spanish–American war [4], influenza during World War I [5], and malaria during World War II and the Vietnam War [6]. Additionally, diseases once collectively described as ‘rheumatism’ that have long plagued military units have since been partly attributed to long-term sequelae resulting from infectious diseases such as malaria, dysentery, and syphilis [7]. The impact of DNBIs is famously exemplified by the pivotal Battle of Valmy during the Prussian invasion of France in 1792, where a Prussian offensive of 34,000 troops was stalled by staggering rates of dysentery compounded by heavy rain and long supply lines, ultimately forcing their retreat from Valmy and eventual withdrawal from France, despite Prussian losses amounting to less than 200 troops during the battle [3]. 

With the advent of public health measures such as sanitation, vector control, and antibiotic/antimalarial drugs, the impact of DNBIs is significantly lower in modern warfare [8]. However, respiratory illness and diarrheal disease, though often mild or routine, occur at relatively high frequency in military populations and still account for a substantial portion of medical encounters and lost duty days [9]. Furthermore, these otherwise effective public health measures are often disrupted during early or intense stages of military operations leading to disease outbreaks, such as in Operation Serval in 2013, where the annual number of diarrhea cases nearly doubled compared to prior and subsequent years (unpublished data from the French Armed Forces Center for Epidemiology and Public Health). 

Respiratory illnesses continue to account for a significant source of lost duty days during military deployments. Etiological agents typically include rhinoviruses, enteroviruses, human coronaviruses, adenovirus, influenza virus, parainfluenza virus, and respiratory syncytial viral (RSV) [10,11,12]. These overall etiologies are largely reflected in the civilian population in Southeast Asia but symptoms can be exacerbated due to climate, poor air quality, or physical exertion and fatigue. Military recruits have been shown to be particularly vulnerable to adenoviruses such as Ad4 [13,14,15]. Most recently, an outbreak of Ad7 among military conscripts in Finland led to the hospitalization of 129 individuals, military and civilian [16]. The emergence of SARS-CoV-2 showed how quickly novel respiratory pathogens can spread in a military population particularly in training environments [17] and on board naval vessels [18]. Finally, in deployment settings, it is largely unknown to what extent respiratory illnesses originate from a unit’s home location and spread internally among the deployed unit, and to what extent they are acquired from the local population.

Diarrheal diseases persist as a significant source of DNBIs in modern military operations, particularly in the Indo-Pacific region [9]. Recent studies with the U.S. military show consistently high attack rates for service members during deployments for military exercises in the area [19]. Enterotoxigenic *Escherichia coli* (ETEC), *Campylobacter*, and *Shigella* are among common etiologic agents. Regional case–control studies in Thailand and Cambodia show high rates of diarrheagenic *E. coli* and *Campylobacter* [20,21] which is also largely reflected in diarrhea studies of Western travelers in the region [22,23]. Finally, in addition to acute gastrointestinal illnesses, enteropathogens such as *Shigella*, *Salmonella*, *Campylobacter*, and *Giardia* have been attributed to latent or chronic rheumatological sequelae such as Reiter’s Disease or reactive arthritis in military personnel [24,25], underscoring both the immediate and second-order impacts of diarrheal illness on military health and readiness. 

For several years, the Walter Reed Army Institute of Research – Armed Forces Research Institute of Medical Sciences (WRAIR-AFRIMS) has been conducting disease surveillance during large annual multi-national military exercises in the region including Cobra Gold in Thailand and Balikatan in the Philippines [26,27,28,29,30,31,32]. With advances in field-capable molecular diagnostics, such as with the BioFire platform [33,34,35], it is now possible to conduct real-time disease surveillance using molecular detection in a field environment. Here, we outline our implementation of this new field-based disease surveillance approach at Cobra Gold and Balikatan exercises from 2023-2025 and highlight key findings including symptom profiles, disease etiologies, and more advanced pathogen characterization such as viral sequencing and bacterial antimicrobial susceptibility testing results. 

## 2. Materials and Methods

**Figure 1:**
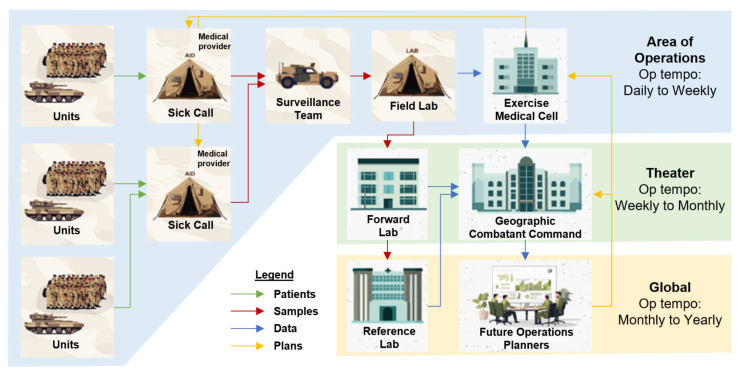
Within the exercise Area of Operations (AO), service members with acute respiratory or diarrheal disease reported to a field aid station, where an HCP requested a biospecimen. The biospecimen was transported by the surveillance team to the Field Lab, located within the AO for sample analysis. Samples were analyzed by PCR daily and results were reported weekly to the Exercise Medical Cell. At the end of the exercise, samples were shipped from the Field Lab to the Forward Lab (WRAIR-AFRIMS in Bangkok, Thailand) where follow-up analyses such as bacterial culture, antimicrobial susceptibility testing, and viral sequencing were conducted. A final report was compiled annually and provided to the Geographic Combatant Command and other stakeholders to support future operations planning. Finally, any samples of interest were shipped to a reference lab in the U.S. (WRAIR in Silver Spring, MD) for further analysis.

WRAIR-AFRIMS conducted diarrheal and respiratory disease surveillance on U.S. military personnel deployed to the annual military exercises Cobra Gold in Thailand and Balikatan in the Philippines from 2023 to 2025. The surveillance was conducted at the request of the Joint Task Force Surgeon for both exercises. The WRAIR Human Subjects Protection Office made the determination that the surveillance activity was not human subject research due to its nature as a public health activity. All participation in the surveillance activity was voluntary. An overview of the surveillance workflow is shown in Figure 1.

### 2.1. Sample Collection

The surveillance team coordinated with military healthcare providers in the field at field aid stations. These locations are typically where routine sick calls are handled by a healthcare provider (HCP), either a physician, physician’s assistant, nurse, or medic. On a weekly basis, the surveillance team would provide sample kits to each aid station that included a symptom questionnaire, a nasopharyngeal (NPA) swab kit, or a stool collection kit. When service members reported to sick call with acute respiratory illness or diarrhea symptoms, the HCP asked the service member for verbal consent to provide a sample and filled out a short symptom survey. Respiratory illness symptoms were defined as including a runny nose, sore throat, or coughing. Diarrheal disease symptoms were defined as having multiple incidents of loose or watery stool in the past 24 h. Case definitions were intentionally loosely defined to minimize effort on the part of the HCP and maximize participation among service members. The surveillance team would check in with each aid station daily and pick up any samples for testing at the field lab with 24 hrs of sample collection. All samples were placed in insulated containers on ice for storage and transport prior to testing. As military units changed locations, surveillance teams made adjustments to maintain a consistent surveillance schedule.

### 2.2. Molecular Testing

The surveillance team set up a field laboratory typically in a field aid station or a semi-permanent structure. This laboratory consisted of cold storage, either a −20 °C freezer or an insulated box with dry ice, a lab bench for sample processing, and a field-ready PCR machine, typically the BioFire® FilmArray 2.0 or the BioFire® FilmArray Torch (bioMérieux, Marcy l’Étoile, France). In CG in 2024–2025 and in BK 2025, samples were run using the BioFire FilmArray Respiratory 2.1 Panel (RP2.1) [33,35] and the FilmArray Gastrointestinal panel (GI) [34] to test on respiratory and diarrheal samples, respectively. In CG 2023 and BK in 2023–2024, samples were run in the WRAIR-AFRIMS laboratory in Manila or Bangkok, rather than in a field laboratory, using the Fast Track Diagnostics Respiratory Pathogen 21 (FTD21) panel [36] (FTD21; Fast Track Diagnostics, Esch-sur-Alzette, Luxembourg) for respiratory samples, and the diarrheal samples were run using the TaqMan Array Card (TAC) panel [37]. After running an aliquot of the sample with PCR, respiratory samples were placed in cold storage. Stool samples were aliquoted into modified Cary-Blair (mCB) transport medium and/or cryopreserved in 15% glycerol stock and as frozen samples depending on the collection location. Results from the PCR testing were collected daily and compiled into a weekly report that was provided to the Force Health Protection officer for the exercise. All stored samples were shipped to WRAIR-AFRIMS labs in Bangkok for follow-up testing. 

### 2.3. Viral Whole-Genome Sequencing (WGS)

Respiratory samples collected from BK in 2025 were subjected to viral WGS using a hybrid-capture approach. DNA library preparation was conducted with the Illumina Viral Surveillance Panel v2 (VSP v2), followed by sequencing on the Illumina MiSeq platform. Raw sequence reads were quality filtered and trimmed using BBTools v38.22 [38] to remove adapter sequences and low-quality bases or reads. The trimming criteria were as follows: trimming quality ≥ 30, minimum read length ≥ 100 bp, and minimum average read quality ≥ 20. Trimmed reads were then mapped to the corresponding reference genomes using BWA v0.7.17-r1188 [39], with the following references: NC_045512.2 (SARS-CoV-2), PQ736589.1 (HPIV-1), OQ990766.1 (HPIV-2), OR728657.1 (HPIV-3), PV660300.1 (HCoV-OC43), ON791801.1 (HCoV-229E), DQ473502.1 (HRV), and EPI359088–EPI359095 (Influenza A/pdm H1N1). 

Consensus sequences were generated using iVar v1.3.1 [40], applying a minimum depth of coverage of 10 reads and a base quality threshold of ≥30. To provide a preliminary of geographic origin, the resulting consensus sequences were queried against the NCBI Virus database [39] using BLAST (v. 2.17.0) to identify the closest reference sequences. Sequencing results were analyzed by running BLAST against the NCBI Virus database [41] to determine the geographic origin of the closest reference sequence. 

### 2.4. Culture Isolation and Antimicrobial Susceptibility Testing (AST)

For all stool samples collected during Cobra Gold (2023–2025) and for a subset of stool samples collected during Balikatan 2025, we carried out stool culture on fresh or cryo-preserved samples. Prior to 2025, stool samples from Balikatan were not stored in CB media for follow-up analysis. Stool samples (in mCB or in glycerol) were resuspended and inoculated into a range of selective media and enrichment broths: MacConkey, Hektoen, thiosulfate citrate bile salts sucrose, modified semisolid Rappaport Vassiliadis, modified charcoal cefoperazone deoxycholate agar, buffered peptone water, alkali peptone water, and Preston selective enrichment broth in order to culture *Aeromonas*, *Arcobacter*, *Campylobacter*, *E. coli*, *Plesiomonas*, *Salmonella*, *Shigella*, *Vibrio* spp., and *Yersinia enterocolitica* as described in [42]. Selective plates were used to determine the presence of colonies resembling *Shigella*, *Vibrio*, *Salmonella*, *E. coli*, *Aeromonas*, *Plesiomonas*, *Yersinia*, *Campylobacter*, and *Arcobacter*. Bacterial isolation and identification were performed using standard microbiological techniques [42]. Diarrheagenic *E. coli* were tested by a multiplex Polymerase Chain Reaction (PCR) to determine the pathotypes: *Enteropathogenic E. coli* (EPEC), *Enteroinvasive E. coli* (EIEC), *Enteroaggregative E. coli* (EAEC), *Enterotoxigenic E. coli* (ETEC), and Shiga toxin-producing *E. coli* (STEC) [42].

All *E. coli*, *Plesiomonas*, *Salmonella*, and *Vibrio* spp. isolates were tested for antimicrobial susceptibility to the following antibiotics by disc diffusion method according to Clinical and Laboratory Standards Institute (CLSI, 2020) guidelines [43]: ampicillin (AMP), azithromycin (AZM), ciprofloxacin (CIP), nalidixic acid (NAL), trimethoprim-sulfamethoxazole (SXT), ceftazidime (CAZ), ceftriaxone (CRO), cefotaxime (CTX), tetracycline (TE), imipenem (IMP), and meropenem (MEM). *Campylobacter* and *Arcobacter* isolates were tested for antimicrobial susceptibility to erythromycin (ERY), AZM, CIP, and NAL by E-test (Liofilchem, Roseto degli Abruzzi, Italy) according to the National Antimicrobial Resistance Monitoring System for Enteric Bacteria [44].

### 2.5. Data Analysis and Reporting

All molecular data collected in the field was compiled into a weekly report and provided to the Joint Task Force or Exercise Force Health Protection officer. Care was taken to ensure that the data remained anonymous and that personally identifiable information could not be obtained from individual cases. Only general location information on pathogens detected was provided and unit names were omitted. Follow-up analysis was compiled into a post-exercise report that was delivered to the exercise medical planners to help guide FHP policy for future iterations of the exercise. The laboratory procedures, such as field molecular testing, were authorized for conducting for public health purposes only and were not used for clinical diagnostic purposes.

## 3. Results

### 3.1. Demographics and Clinical Findings

Over a three-year period from 2023–2025, we collected 84 respiratory samples and 61 diarrheal samples in the annual Cobra Gold exercise in Thailand and the Balikatan exercise in Philippines. We observed an increase in participation in the study over the three years in both exercises. As expected, the population was highly skewed to younger ages and male gender, with 68% of subjects under 30 y.o. (90% under 40) and 91% of subjects being male (Table 1). 

**Figure 2:**
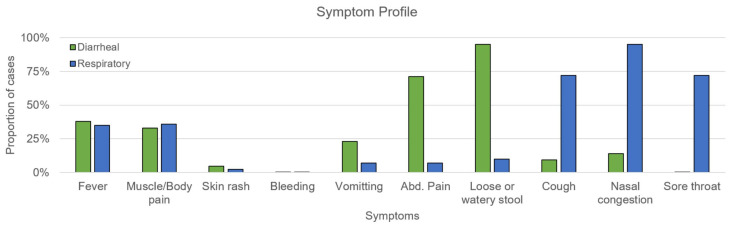
Symptom profile for diarrheal (green) and respiratory (blue) cases for CG and BK from 2023–2025 as a proportion of total number of cases in each category.

Results from the symptom survey reflected both non-specific and specific symptoms associated with respiratory illness and diarrheal disease (Figure 2). Fever and muscle or body pain were found at similar rates for both diarrheal and respiratory cases at 30–40%. Diarrheal disease was specifically associated with high rates of abdominal pain (71%) with a low to moderate rate of vomiting (24%). Respiratory cases were associated with high rates of nasal congestion (95%), cough (72%), and sore throat (72%).

### 3.2. Respiratory Illness Etiology

**Figure 3:**
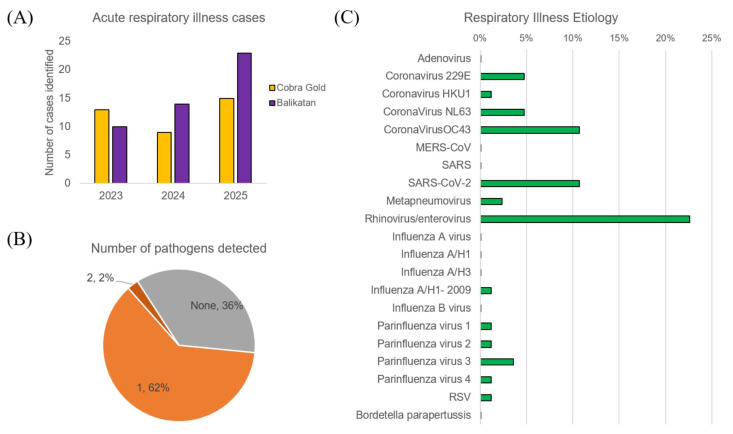
Respiratory illness etiology in CG and BK from 2023–2025 as identified through field molecular testing: (**A**) Number of accurate respiratory illness cases identified from 2023–2025 for CG (gold) and BK (purple) (**B**) The percentage of samples in which zero, one, or two pathogens were detected. (**C**) The proportion of cases in which bacterial (blue), parasitic (yellow), and viral pathogens (green) were identified.

We saw a moderate increase in participation in the surveillance activity from 23 cases in 2023 to 38 cases in 2025 (Figure 3). In terms of identifying an etiological agent through field molecular testing, no pathogen could be identified in 36% of the samples, 62% of the samples had one pathogen identified, and 2% of the samples had two pathogens identified. Our pathogen panels included both bacterial and viral pathogens but only viral pathogens were detected. The most common respiratory pathogens identified were rhinoviruses/enteroviruses (23%), common coronaviruses (21%), SARS-CoV-2 (11%), and parainfluenza viruses (7%). We also detected two cases of metapneumovirus, one case of RSV, and one case of influenza A.

Viral WGS was successfully performed on 24 respiratory samples collected during BK 2025. All PCR-positive detections were confirmed by WGS, demonstrating strong concordance between PCR and sequencing-based methods. A single discrepancy was observed in one sample, where the BioFire^®^ assay initially detected both SARS-CoV-2 and HCoV-OC43, while WGS identified only HCoV-OC43. Repeat testing at WRAIR-AFRIMS confirmed the latter finding, emphasizing the importance of multi-platform verification. BLAST analysis against the NCBI database (Table 2) showed that SARS-CoV-2 and Influenza A/pdm H1N1 sequences shared greater than 99.9% genomic similarity with U.S. reference strains, suggesting close genetic relatedness and potential importation or shared transmission pathways. Similarly, HCoV-OC43, HCoV-229E, and HPIVs sequences clustered closely with global reference strains from the U.S. and Asia, indicating regional co-circulation of these respiratory viruses. The consensus sequences generated in this study have been submitted to the GenBank database and are available upon request.

### 3.3. Diarrheal Disease Etiology

**Figure 4:**
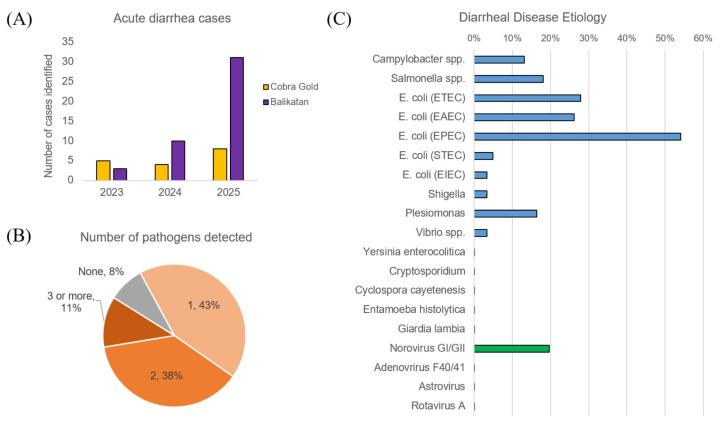
Diarrheal etiology in CG and BK from 2023–2025 as identified through field molecular testing: (**A**) Number of diarrheal disease cases identified from 2023–2025 for CG (gold) and BK (purple). (**B**) The percentage of samples in which zero, one, two, and more than two pathogens were detected. (**C**) The proportion of cases in which bacterial (blue), parasitic (yellow), and viral pathogens (green) were identified.

We saw a steady increase in participation in diarrheal surveillance from 8 total cases in 2023 to 39 total cases in 2025 (Figure 4A). Overall, when identifying diarrheal disease etiology using molecular testing, we found that 8% of the cases had no potential pathogen identified, in 43% of the cases one pathogen could be identified, and in 42% multiple potential pathogens were identified.

The types of pathogens could be broadly classified as bacterial, parasitic, and viral. We found that bacterial pathogens accounted for a majority of the samples, with diarrheagenic *E. coli* as the most prevalent (72%), particularly EPEC (54%), ETEC (28%), and EAEC (26%). In addition to diarrheagenic *E. coli*, we observed *Salmonella* spp. (18%), *Plesiomonas* (16%), *Campylobacter* (13%), and *Shigella* spp. (3%). Among viral pathogens, we found a relatively high rate of norovirus (20%).

Stool samples were preserved and transported to our central laboratory in Bangkok for culturing. We found that recovery rate varied by pathogen in terms of being able to culture an isolate of the same pathogen type that was detected in the sample by PCR. For diarrheagenic *E. coli*, the recovery rate was 56%, compared to 57% for *Campylobacter* spp. and 33% for *P. shigelloides*. We found 100% recovery rates for *Salmonella* spp. and *Vibrio cholera* (non O1/O139). When using the stool culture as a reference for comparison, the field PCR achieved a sensitivity of 82% and a specificity of 88% for detecting bacterial enteropathogens that were identified by stool culture. For specimens that we were able to successfully culture, we carried out AST using the disk diffusion method. Table 3 has results on the AST results. Resistance patterns varied by diarrheal pathogen, but we found resistance to AMP, NAL, TE, and SXT (also known as Bactrim) across multiple diarrheal bacterial pathogens including EPEC, EAEC, *Salmonella*, and *P. shigelloides*. For *Campylobacter* and *P. shigelloides*, we observed resistance to AZM, CIP, and NAL acid. Finally, for *Campylobacter* spp., we also observed resistance to ERY.

## 4. Discussion

Here, we describe our efforts to conduct respiratory and diarrheal disease surveillance with U.S. military personnel that deployed to the annual Cobra Gold and Balikatan military exercises in Thailand and the Philippines from 2023 to 2025. The goal of the effort was two-fold: (1) to identify etiological agents responsible for acute respiratory or diarrheal disease in U.S. personnel during military exercises in Southeast Asia and (2) to demonstrate the feasibility of conducting real-time field molecular testing as part of disease surveillance in military operational settings. 

For respiratory illnesses identified during the BK 2025 investigation, the primary etiological agents were human rhinoviruses and enteroviruses, followed by common human coronaviruses, SARS-CoV-2 (COVID-19), and parainfluenza viruses. This pathogen distribution closely mirrors patterns reported in civilian populations across Southeast Asia [45,46], where studies on acute lower respiratory infections consistently identify rhinovirus/enterovirus as the most prevalent agents, with lower detection rates for human coronaviruses and parainfluenza viruses. However, in contrast to those studies in civilian cohorts, RSV and influenza viruses appeared less frequently in this military population. Such differences in pathogen prevalence may be attributable to factors such as the highly skewed demographics or high vaccination rates of deployed military personnel. 

In deployed military settings, a key question is whether circulating respiratory pathogens are introduced from local populations or carried from personnel’s home stations. WGS provided important insights into this issue during BK 2025. Preliminary BLAST analysis revealed that the majority of viral genomes (83%) were most closely related to reference strains from North America, while 17% aligned with Asian strains. These findings suggest that most respiratory pathogens were likely imported from the troops’ home stations and subsequently propagated through person-to-person transmission within deployed units, rather than being newly acquired from host-nation personnel or local com-munities, consistent with observations of multiple introductions and local transmission in Southeast Asia [47]. It is important to emphasize that BLAST-based similarity analyses provide only a preliminary view of genetic relatedness and potential geographic origin. A deeper understanding of viral transmission, introduction events, and evolutionary history requires robust phylogenetic and phylodynamic analyses. These approaches can identify independent introductions, estimate the timing of viral diversification, and clarify transmission pathways within and between deployed units [48]. The highly interactive and confined living conditions of deployed military environments where personnel train, sleep, and eat together, likely facilitate rapid intra-unit transmission once pathogens are introduced. These findings highlight the critical need for ongoing genomic surveillance, pre-deployment screening, and targeted vaccination strategies to prevent both the introduction and sustained spread of respiratory pathogens during multinational military exercises.

For diarrheal disease, we found that the etiological agents were predominantly bacterial, mainly diarrheagenic *E. coli* (EPEC, ETEC, and EAEC). However, *Plesiomonas*, *Salmonella*, and *Campylobacter* also accounted for a significant number of cases. We also observed a relatively high rate of norovirus (20%). In a recent case–control study in neighboring Cambodia [21], we found that, while diarrheagenic *E. coli* was also the most common etiological agent in civilian population, rates of *Campylobacter* and norovirus in the adult civilian population were much lower (2% and 1%, respectively) [21] than what we observed here (13% and 20%, respectively), suggesting that deployed military personnel may have higher susceptibility to these pathogens. It is important to note that the overall prevalence of *Campylobacter* was lower than expected and may reflect a larger etiological shift in SE Asia. In diarrhea studies conducted in the region prior to 2015, *Campylobacter* was the most common etiological agent in both civilian [20] and military/traveler populations [23,27,30], accounting from 20-35% of cases, while the case–control study in the civilian population conducted in 2020-2023 found that *Campylobacter* accounted for only 1% to 5% of acute diarrhea cases, depending on age group [21]. One possible explanation could be regional differences in *Campylobacter* prevalence within SE Asia, but we did not observe a notable difference in *Campylobacter* prevalence between acute diarrhea cases from BK (Philippines) and CG (Thailand) in this study. Furthermore, prior studies in U.S. military personnel deployed specifically to CG in 2002-2003 [27] and in BK in 2014 [30] in similar locations as the present study both identified *Campylobacter* as the most prevalent pathogen in acute diarrhea cases, suggesting this apparent etiological shift is not simply a result of study-specific differences in regions or populations.

We achieved a recovery rate of 50 to 100% in terms of successfully culturing an isolate of the same pathogen type that was detected by field PCR in a sample for most bacterial enteropathogens we tested for, indicating that sample integrity was not significantly compromised in a field environment. Assessing antimicrobial resistance is critical to determining whether existing treatment guidelines remain effective. We assessed the AST profile of all cultured isolates and found high rates of resistance to ampicilin and trimethoprim-sulfamethoxazole (Bactrim), and low-to-moderate rates of resistance to quinolones (ciprofloxacin and nalidixic acid) and azithromycin across multiple bacterial enteric pathogens. We observed high resistance in *Campylobacter* to quinolones and moderate resistance to azithromycin and erythromycin, corroborating what has been observed in Southeast Asia [21,27,49], in contrast to other regions where *Campylobacter* spp. remain largely sensitive to quinolones and other antibiotics [50]. There is a high rate of antibiotic use in the civilian population in SE Asia, and Bactrim is the most commonly locally prescribed antibiotic of diarrhea [21]. By contrast, Western travelers and military personnel are often provided with azithromycin or ciprofloxacin. Antibiotics use guidelines for treating acute diarrhea in SE Asia which have long accounted for ciprofloxacin resistance, particularly in *Campylobacter* and *Shigella*, with azithromycin being recommended as the first-line treatment [51]. However, recent findings both in this study and elsewhere [21] suggest that these guidelines may have to be updated to account for an increase in azithromycin resistance compared to previous years [27].

Overall, we found that we could feasibly conduct disease surveillance using field-based molecular testing in a military operational setting, but that challenges remain. In Balikatan 2025, for instance, our surveillance observed an increase in the number of norovirus cases that coincided with an increase in diarrhea incidence rates during sick call as reported to us anecdotally by HCPs at the time. However, in another instance, we were not as successful. Also in Balikatan 2025, there was an outbreak of 28 diarrhea cases reported over the span of 72 hrs (~30% attack rate) in a remote location in Northern Luzon, Philippines. We were able to send sample kits to that location by helicopter but had to retrieve the specimens by ground transport. By the time the samples arrived at the field laboratory, they were several days old and had degraded and testing results were inconclusive. 

We identified several areas for improvement in our surveillance effort. First, we found that participation rates varied significantly by location and provider, underscoring the importance of engaging with providers and communicating the importance of the surveillance activity. Second, we observed a high reluctance to provide fresh stool samples in personnel that presented with acute diarrhea symptoms, suggesting that alternate fecal sample collection methods may be necessary such as rectal swabs [52] or wipe-based collection kits [53]. Third, we found that the transport, storage, and timely testing of fresh stool specimens in an operational environment was challenging, and the use of a nucleic acid stabilizing transport media that extends the stability and durability of samples for field testing should be explored. Finally, fourth, we encountered significant logistical challenges with keeping tracking unit positions, resupplying providers, and ensuring that air transport was available for transport to remote locations, highlighting the importance of better coordination with medical units on the ground for both routine sample collection and outbreak response. 

## 5. Conclusions

Here, we demonstrate the ability to carry out respiratory and diarrheal disease surveillance of deployed military personnel in operational settings. We were able to characterize the etiologies of diarrheal and respiratory illness using field molecular testing and found evidence for increased susceptibility in the deployed military population for diarrheal pathogens such as *E. coli*, ETEC and EPEC, *Campylobacter*, and norovirus. We also found, surprisingly, that respiratory pathogens in these deployed personnel appear to originate largely from their home stations, rather than being newly acquired through interactions with host-nation military personnel or the local population. Our findings highlight the value of conducting disease surveillance in deployed military personnel and provide a framework for conducting future biosurveillance activities as part of large annual multi-national military exercises.

## Figures and Tables

**Table 1:** Demographics and sample numbers.

	Cobra Gold 2023–2025	Balikatan 2023–2025
Total Cases	54	91
		
Respiratory Illness Cases	37 (69%)	48 (53%)
Diarrheal Disease Cases	17 (31%)	43 (47%)
		
<30 years old	37 (69%)	60 (66%)
30 to 39 years old	11 (20%)	22 (24%)
40 to 49 years old	5 (9%)	2 (2%)
≥50 years old	1 (2%)	6 (7%)
		
Male	40 (93%)	82 (90%)
Female	5 (7%)	9 (10%)

**Table 2:** Geographic origin of respiratory viral pathogens in BK 2025.

Pathogen	*N*	Geographic Origin of Nearest Reference Sequence
Rhinoviruses	1	North America (1)
Enteroviruses	0	
Coronavirus 229E	1	North America (1)
Coronavirus HKU1	0	North America (1)
Coronavirus NL63	0	
Coronavirus OC43	4	North America (4)
SARS-CoV-2	1	North America (1)
Influenza A/pdm H1N1	1	North America (1)
Parainfluenza 1	1	Asia (1)
Parainfluenza 2	1	Asia (1)
Parainfluenza 3	2	North America (2)
Parainfluenza 4	0	
No pathogen detected	12	

**Table 3:** Antibiotic susceptibility testing results.

Pathogen	*N*	AMP	AZM	CIP	NAL	ERY	SXT	CAZ	CRO	CTX	TE	IMP	MEM
*Campylobacter coli*	2		50%	100%	100%	50%							
*Campylobacter jejuni*	3		33%	100%	100%	0%							
EPEC	7	71%	14%	0%	0%		29%	0%	14%	14%	71%	14%	0%
ETEC	3	0%	0%	0%	0%		0%	0%	0%	0%	0%	0%	0%
EAEC	6	67%	17%	17%	17%		17%	0%	0%	0%	83%	0%	0%
*Plesiomonas shigelloides*	3	33%	33%	33%	67%		67%	0%	0%	0%	0%	0%	0%
*Salmonella* spp.	10	60%	0%	10%	20%		20%	10%	10%	10%	60%	0%	0%
*Vibrio cholera (non O1/O139)*	1	0%	0%	0%	100%		0%	0%	0%	0%	0%	0%	0%

## Data Availability

All data are contained within the manuscript.

## Abbreviations

The following abbreviations are used in this manuscript:

PCR	Polymerase Chain Reaction
HCP	Healthcare Provider
EPEC	*Enteropathogenic E. col*
EIEC	*Enteroinvasive E. coli*
EAEC	*Enteroaggregative E. coli*
ETEC	*Enterotoxigenic E. coli*
STEC	Shiga toxin-producing *E. coli*
AMP	ampicillin
AXM	azithromycin
CIP	ciprofloxacin
NAL	nalidixic acid
SXT	trimethoprim-sulfamethoxazole
CAZ	ceftazidime
CRO	ceftriaxone
CTX	cefotaxime
TE	tetracycline
IMP	imipenem
MEM	meropenem
ERY	erythromycin
RSV	respiratory syncytial viral
NPA	nasopharyngeal
AST	Antimicrobial susceptibility testing
CG	Cobra Gold
BK	Balikatan
WRAIR	Walter Reed Army Institute of Research
WRAIR-AFRIMS	Walter Reed Army Institute of Research—Armed Forces Research Institute of Medical Sciences
